# Exertional intolerance and dyspnea with preserved lung function: an emerging long COVID phenotype?

**DOI:** 10.1186/s12931-021-01814-9

**Published:** 2021-08-06

**Authors:** Grace Y. Lam, A. Dean Befus, Ronald W. Damant, Giovanni Ferrara, Desi P. Fuhr, Michael K. Stickland, Rhea A. Varughese, Eric Y. Wong, Maeve P. Smith

**Affiliations:** 1 Division of Pulmonary Medicine, Department of Medicine, University of Alberta and Alberta Health Services, 3-111C Clinical Sciences Building, 11302 83 Ave NW, Edmonton, AB T6G 2G3 Canada; 2 Alberta Respiratory Centre, University of Alberta, Edmonton, AB Canada

**Keywords:** Long COVID, Post-acute sequelae of COVID-19, Long-haulers

## Abstract

The COVID-19 pandemic has resulted in significant acute morbidity and mortality worldwide. There is now a growing recognition of the longer-term sequelae of this infection, termed “long COVID”. However, little is known about this condition. Here, we describe a distinct phenotype seen in a subset of patients with long COVID who have reduced exercise tolerance as measured by the 6 min walk test. They are associated with significant exertional dyspnea, reduced health-related quality of life and poor functional status. However, surprisingly, they do not appear to have any major pulmonary function abnormalities or increased burden of neurologic, musculoskeletal or fatigue symptoms.

## Research Letter

Coronavirus-19 (COVID-19) caused by severe acute respiratory syndrome coronavirus-2 (SARS-CoV-2) has rapidly spread across the globe, resulting in significant morbidity and mortality. While much focus has been paid to the acute phase of infection, there is a growing recognition of the longer-term sequelae of COVID-19, or “long COVID”, defined by many as persistent post COVID-19 symptoms lasting beyond 12 weeks post infection [1]. While long COVID is thought to affect 10–50% of COVID-19 survivors [2, 3], little is known about who might develop these complications, which risk factors might predispose their development or how long they can persist.

Current literature suggests that patients with long COVID experience significant multi-system symptoms [4, 5]. These complications have been reported in patients who had severe acute COVID-19 infections (requiring hospitalization) as well as those who had mild infections (not requiring hospitalization). In COVID-19 survivors of severe acute infections, the majority were found to have persistent fatigue, breathlessness or significant burden of psychological distress and reduced health-related quality of life at 1–2 months post discharge [6, 7], while 63% still reported persistent fatigue or muscle weakness at 6 months’ follow up [8]. Additionally, the 6-min walk test (6MWT), a simple but powerful tool that reflects exercise tolerance, was decreased in one-quarter of this cohort, independent of the care they received in hospital (routine hospitalization versus intensive care) [8]. This is a particularly striking finding given that the median age of patients in this study was 57 years.

To better understand this subset of patients with reduced exertional tolerance (defined as those with walk distance lower than their calculated lower limit of normal based on age, sex, weight and height) [9], we examined the first 165 patients seen in our Post COVID-19 Clinic in Alberta, Canada (June 2020–April 2021). Patients with persistent symptoms of at least 4 weeks in duration following acute COVID-19 are accepted for referral, regardless of the severity of their initial infection. The study was approved by the University of Alberta Research Ethics Board (Pro00104564) and conducted in full accordance with the ethics board guidelines. Statistical analyses were done with Prism 9.0 using non-parametric Mann–Whitney test unless otherwise specified.

**Fig. 1:**
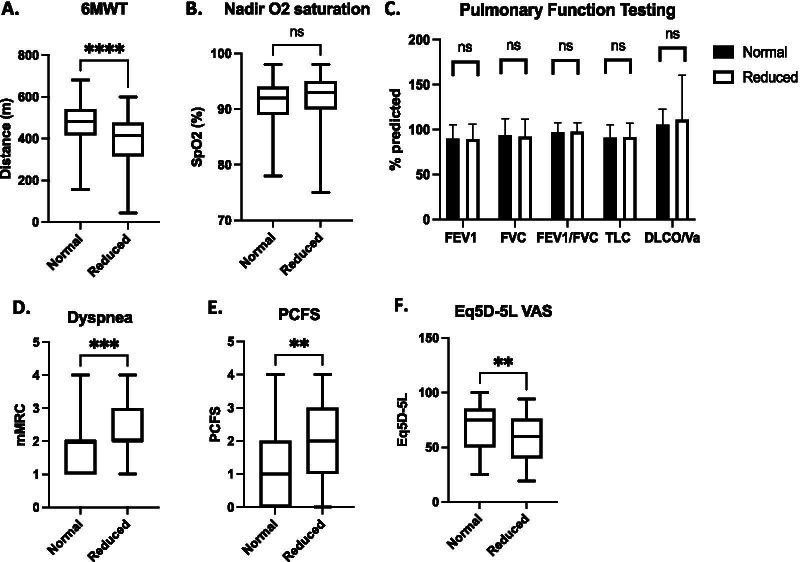
Characterizing Patients with Long COVID who had Normal 6-min walk test (6MWT) Distances Versus Those who had Reduced Distances (Total n = 165) by **A** 6MWT Distance; **B** Oxygen Saturation Nadir; **C** Pulmonary Function Testing; **D** Modified Medical Research Council (mMRC) Subjective Dyspnea Score; **E** Post-COVID Functional Scale (PCFS) score; **F** Health-related Quality of Life (EQ–5D–5L) score

The demographics (mean [standard deviation]) of these 165 patients are as follows: age 50.7[16]; body mass index (BMI) 30.7[7.4]; modified Medical Research Council (mMRC) dyspnea score 2.0[0.8]; health-related quality of life metric EQ–5D–5L 65[20.6] and days from first molecular test positivity 121.6[38.4]. 66 of the 165 patients (40%) required hospitalization during acute infection. 59 of the 165 patients (36%) had a 6MWT distance less than the lower limit of normal (Fig. 1A). Of the patients who had reduced exertional tolerance: age 40.3[14.8]; BMI 28.9[6.9]; and days from molecular test positivity 112.0[65.4]. Of the patients who had normal exertional tolerance: age 53.7[13.8]; BMI 30.7[7.4]; and days from molecular test positivity 136.5[68.4]. Unexpectedly, patients with reduced exertional tolerance were statistically younger (40.3[14.8] versus 56.5[13.6]; *p* < 0.0001) and lighter (28.9[6.9] versus 31.7[7.6]; *p* = 0.02) than the patients with normal exertional tolerance. With regards to pre-existing comorbidities, patients with normal exertional tolerance had a significantly higher rate of previous or active smoking (11/59 vs 37/106; *p* = 0.03) and hypertension (7/52 vs 34/106; *p* = 0.004) but otherwise no differences were found regarding rates of marijuana use or vaping, asthma, chronic obstructive pulmonary disease, coronary artery disease, congestive heart failure, diabetes, mental health disorders, musculoskeletal disease or peripheral vascular disease. No other statistically significant differences in baseline demographics between the two groups were noted. The rates of severe acute COVID-19 requiring hospitalization (35% (20/59) versus 43% (46/106)) and ICU admission (7% (4/59) versus 16% (17/106) were similar in both groups. With regards to treatment while in hospital, less patients received oxygen therapy (20% (12/59) vs 41% (43/106); *p* = 0.008) or dexamethasone (20% (12/59) vs 41% (44/106); *p* = 0.008) in those with reduced exertional tolerance than those with normal exertional tolerance. There were minimal use of remdesivir (0/59 vs 3/106) and hydroxychloroquin (0/59 vs 2/106) in both groups. Assessment of patient reported mental health scores, those with reduced exertional tolerance reported a higher Patient Health Assessment-9 score (PHQ-9; validated questionnaire of depression) than those with normal exertional tolerance (10[6.9] vs 6.7[5.7]; *p* = 0.01) but similar Generalized Anxiety Disorder-7 score (GAD-7; validated questionnaire of anxiety) in both groups (6.8[6.1] vs 5.3[5.5]; *p* = 0.2). 2 patients in the whole cohort (both with normal exertional tolerance) had attended or were attending rehabilitation or physiotherapy programs prior to being seen in the clinic.

The nadir oxygen saturation observed during the walk test was similar between those with normal versus reduced exertional tolerance (Fig. 1B). Overall, lung function on average was within expected normal limits and no differences in forced expiratory volume in one second (FEV_1_), forced vital capacity (FVC), FEV_1_/FVC, total lung capacity (TLC) or diffusion capacity adjusted for alveolar volume (DLCO/VA) between the two groups were noted (Fig. 1C). Chi^2^ analysis of symptoms demonstrated no differences between the two groups with regards to neurologic (*p* = 0.75), musculoskeletal (*p* = 0.14) or fatigue (*p* = 0.17) complaints. Interestingly, subjective mMRC dyspnea score was significantly higher for the reduced exertional tolerance group (Fig. 1D). These patients also reported more functional impairment, as reflected by a higher Post-COVID Functional Scale (PCFS) score (Fig. 1E), and a lower health-related quality of life (EQ–5D–5L) score (Fig. 1F). 69% of patients with normal exertional tolerance reported having returned to work at 3 months post-recovery compared to 62% of patients with reduced exertional tolerance (*p* = 0.53). Collectively, these observations suggest that the difference in walk distance is associated with significant subjective dyspnea and may have significant ramifications on overall function and quality of life but is unlikely to be due to a pulmonary limitation to exercise or confounding neurologic, musculoskeletal or fatigue symptoms.

From these findings, a distinct subset of patients with long COVID has emerged. These individuals may be younger, have marked reductions in exercise tolerance, are functionally limited and have significantly reduced health-related quality of life. Despite being, on average, 3–4 months from acute recovery, they have not yet returned to their previous functional baseline or level of health with a trend towards being less able to return to work. Even those with less severe acute disease not requiring hospitalization appear to be at risk. Given the burden of symptoms, impairment and potential for long-term disability, early identification of these individuals for targeted symptom management, intensive rehabilitation, characterization of disease trajectory and ongoing clinical research is warranted.

What is less clear is the underlying mechanism(s) of exertional limitation. These individuals do not appear to demonstrate pulmonary limitation to exercise, nor are they disproportionately symptomatic from a neurologic, musculoskeletal or fatigue perspective. This could be due to the lack of sensitivity in baseline clinical and pulmonary functional assessments. More sophisticated testing, such as cardiopulmonary exercise test (CPET), may be needed to better understand the cause of the exertional limitation. Alternatively, other factors, including cardiac or vascular dysfunction, mental health comorbidities, or persistent systemic inflammatory response may be contributing to the observed exertional intolerance and dyspnea. The difference in PHQ-9 score between the groups suggests that mental health symptoms might contribute to reduced exertional tolerance. Further cardiac, radiographic, biochemical and pathologic assessment of these individuals will be needed to determine the pathophysiology of exertional limitations in patients with long COVID.

Intriguingly, our finding that those with reduced exertional tolerance had less exposure to dexamethasone raises the possibility that perhaps acute treatment with steroids could help to reduce long COVID complications. Further work with larger numbers and regression analyses controlling for covariates will be required to conclude whether acute use of steroids can independently influence long COVID development.

Patients with long COVID appear to be a heterogeneous population potentially classifiable into distinct phenotypic subgroups. Our findings highlight the importance of the 6MWT as a low cost but critical tool for the identification of individuals with exertional intolerance and dyspnea despite normal lung function.

## Data Availability

The datasets used and analysed during the current study are available from the corresponding author on reasonable request.
